# Transmission of *Opisthorchis viverrini*, *Schistosoma mekongi* and soil-transmitted helminthes on the Mekong Islands, Southern Lao PDR

**DOI:** 10.1186/s40249-017-0343-x

**Published:** 2017-09-04

**Authors:** Youthanavanh Vonghachack, Peter Odermatt, Keoka Taisayyavong, Souphanh Phounsavath, Kongsap Akkhavong, Somphou Sayasone

**Affiliations:** 1 Swiss Tropical and Public Health Institute, Basel, Switzerland; 2 University of Basel, Basel, Switzerland; 3 Faculty of Basic Sciences, University of Health Sciences, Vientiane Capital, Lao PDR; 4 Malariology, Parasitology and Entomology Station, Champasack province, Lao PDR; 5 Provincial Health Department, Champasack province, Lao PDR; 6 National Institute of Public Health, Ministry of Health, Vientiane Capital, Lao PDR

**Keywords:** *Opisthorchis viverrini*, *Schistosoma mekongi*, Animal hosts, *Bithynia* species., *Neotricula aperta*, *Cyprinidae* fish, Southern Lao People's Democratic Republic, Laos

## Abstract

**Background:**

Prevalence of *Opisthorchis viverrini, Schistosoma mekongi* and soil-transmitted helminths (STH) remains high in Lao People’s Democratic Republic (Lao PDR), despite control efforts including mass-drug administration, education and communication campaigns. New approaches are required to advance helminth control.

**Methods:**

An ecohealth study was conducted on two Mekong islands in Southern Laos. Demographic and behavioural data were collected by questionnaire. Human and animal reservoir stools were examined. *Bithynia* spp. and *Neotricula aperta* snails were examined using shedding. Fresh water fish were examined using digestion technique. Multivariate random-effects analysis was used to find risk factors associated with helminth infections.

**Results:**

Human infection rates with *O. viverrini,* hookworm, *S. mekongi, Trichuris trichiura*, *Ascaris lumbricoides* and *Taenia* spp. were 60.7%, 44.1%, 22.2%, 4.1%, 0.6% and 0.1%, respectively. Heavy intensity infections were 4.2%, 3.6% and 1.8% for *O. viverrini*, *S. mekongi* and hookworm, respectively*. O. viverrini* and *S. mekongi* infection rates among dogs and cats were 25.0% and 14.7%, respectively. Of the cats tested, 53.1% were infected with *O. viverrini*. Prevalence of *O. viverrini* and *S. mekongi* in snails was 0.3% and 0.01%, respectively. Overall prevalence of *O. viverrini* infection in fresh water fish was 26.9%, with the highest infection rates occurring in *Hampala dispa* (87.1%)*, Cyclocheilichthys apogon* (85.7%) and *Puntius brevis* (40.0%). Illiteracy and lower socioeconomic status increased the risk of *O. viverrini* infection, while those aged 10–16 years and possessing latrines at home were less likely to be infected. Household dogs and cats that consumed raw fish were significantly and positively associated with *O. viverrini* infection of the household members. For *S. mekongi*, children under 9 years old were exposed significantly to this infection, compared to older age groups.

**Conclusions:**

There is a pressing need to design and implement an integrated helminth control intervention on the Mekong Islands in southern Lao PDR. Given the highly dynamic transmission of *O. viverrini*, *S. mekongi*, STH and extended multiparasitism, annual mass-drug administration is warranted along with environmental modifications, health education and improved access to clean water and adequate sanitation to consolidate morbidity control and move towards elimination.

**Trail registration number:**

Our findings presented here are from a cross-sectional study, therefore, it has not been registered.

**Electronic supplementary material:**

The online version of this article (doi:10.1186/s40249-017-0343-x) contains supplementary material, which is available to authorized users.

## Multilingual abstract

Please see Additional file 1 for translations of the abstract into the five official working languages of the United Nations.

## Background

Helminthiases are neglected tropical diseases (NTDs) of major public health concern in many low- and middle-income countries (LMIC) in the tropics and sub-tropics, including in Lao People’s Democratic Republic (Lao PDR) [1–4]. Liver flukes (*Opisthorchis viverrini),* blood flukes (*Schistosoma mekongi*) and soil-transmitted helminths (STH) such as round worm (*Ascaris lumbricoides*), whipworm (*Trichuris trichiura*) and two-hookworm species (*Ancylostoma duodenale, Necator americanus*) are among the most prevalent infections in Lao PDR. *O. viverrini* is endemic nationwide but is most prevalent in the central and southern parts of the country. It occurs in the lowlands, along the Mekong River, where fish are abundant and local inhabitants prefer to consume traditional dishes prepared with raw fish [1, 4–6]. *S. mekongi* is only endemic in two districts of the most southern province, Champasack, bordering Cambodia [7–10]. STH are highly prevalent in the northern part of the country and in the mountainous areas along Lao-Vietnamese border [4, 11].

Infections with these helminths negatively affect human health and wellbeing. For example, untreated or chronic infection with *O. viverrini* may lead to severe hepatobiliary morbidity including cholangiocarcinoma (CCA), a fatal bile duct cancer [12, 13]. Chronic infection with *S. mekongi* may result in portal hypertension and is associated with peri-portal liver fibrosis [14–17]. In Champasack Province, *O. viverrini* and *S. mekongi* are co-endemic [5, 7, 18], further increasing the risk of hepatobiliary morbidity. Finally, anaemia and undernourishment are associated with long-lasting STH infections [19, 20].

Helminths have complex life cycles; *O. viverrini*, for example, involves two aquatic intermediate hosts, namely freshwater snails (of the genus *Bithynia*) and freshwater fish (of the Cyprinidae family). Humans and other mammals are infected by eating raw or undercooked fish [21]. The life cycle of *S. mekongi* involves humans and other mammals (such as dogs, pigs and possibly rats) [22, 23]. The *Neotricula aperta* snail, which lives in the crevices of submerged rocks in the Mekong River, serves as intermediate host. The cercariae emerge from the infected snails during the daytime and lie under the water surface [9, 24]. Humans and animals are infected with this parasite via skin penetration when they come into contact with infested waters [8]. Lao PDR adheres to the preventive chemotherapy control strategy promoted by WHO [3, 25, 26]. Over the last decade, considerable efforts were employed to implement this strategy through deworming programmes targeting school-children [27] and through mass-drug administration (MDA) alongside information, education and communication (IEC) campaigns in high risk provinces of the country [28]. Despite these efforts, the prevalence of helminth infections, including multiple infections, remains high in many places [4, 18, 26, 29–31]. Given the complexity of the transmission cycle of helminth infections and the risky behaviour of humans in endemic communities, it may be necessary to adapt the control strategy to improve the effectiveness of interventions.

Ecohealth research is an emerging field of research studying human health in close connectivity with the ecosystem [32]. It is increasingly conducted to strengthen the sustainability of infectious disease control programmes [33–35] and was widely introduced in Southeast Asia (SEA) by the Canadian International Development Research Centre (IDRC) in the late 2000s [36, 37]. Ecohealth has been defined as follows: i) “EcoHealth involves research and practice to promote sustainability of individuals, animals and biodiversity by linking complex interaction of ecosystem, socio-cultural and economic factors” and ii) “Ecohealth is a comprehensive approach to understanding health at its human, animal and environmental interface in a socio-ecological systems context”. Here, we employ an ecohealth approach to determine the prevalence and risk factors of *O. viverrini*, *S. mekongi* and STH infections in humans in the ecological environment of Khong district, where potential animal reservoir and intermediate hosts, like molluscs and fish, live in close connectivity.

## Methods

### Study area

**Fig. 1:**
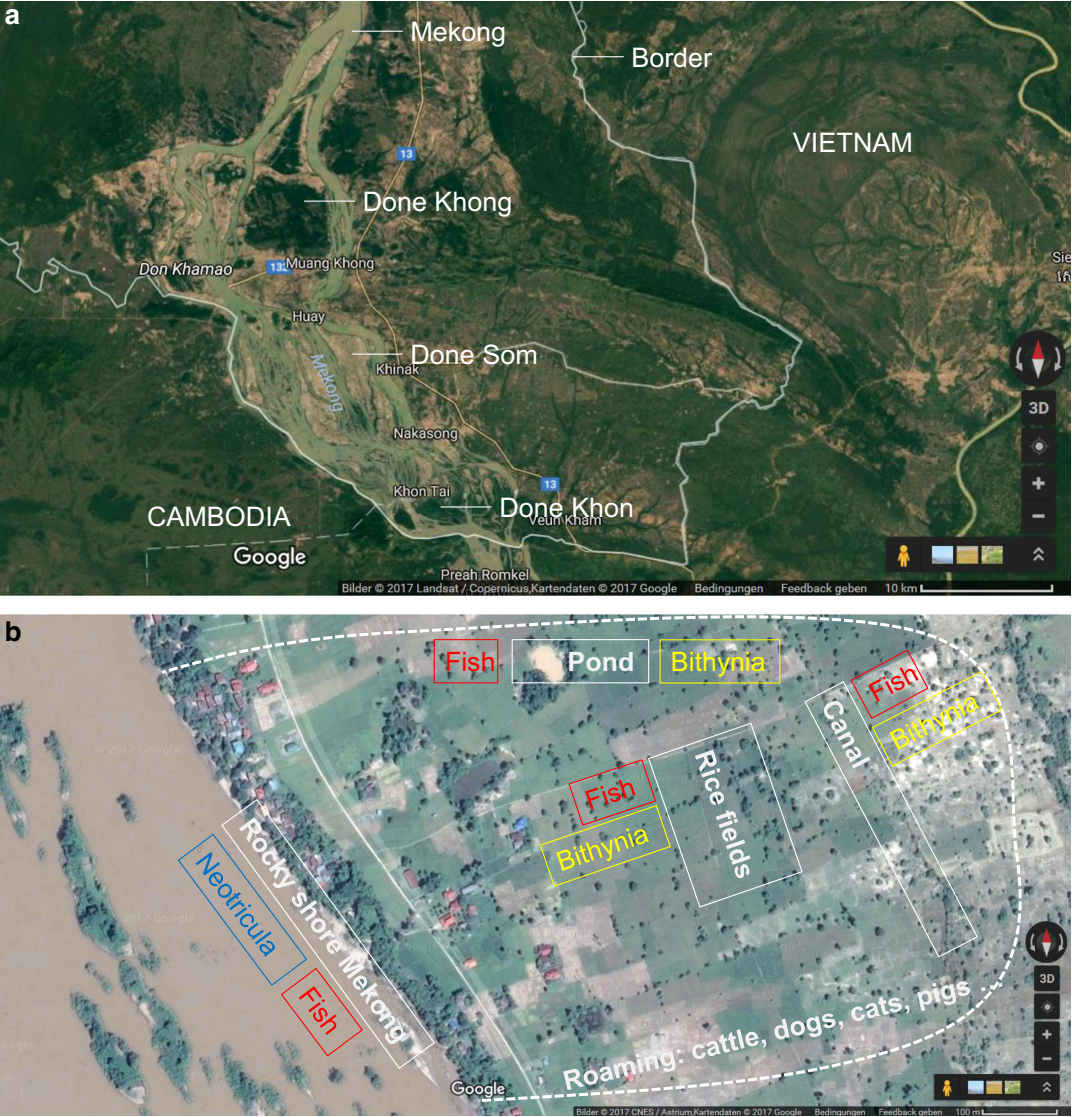
Study map: **a** Khong District with main Mekong islands; **b** Selected western shore of Done Som with human settlements and ecological features. (Source: Google Map)

Khong district is an island district located at the Southern border of Champasack Province, Lao PDR (Fig. 1a). It has an estimated population of 100,000 people and comprises a few dozen islands in the Mekong River (geographical coordinates: 13.57°-14.14°N latitude and 105.44°-106.08°E longitude). The district is a known endemic area for *O. viverrini, S. mekongi* and STH. Done Khon and Done Som are among the biggest islands and are popular tourist destinations. Done Khon has about 260 households with a total population of 1560 people, while Done Som has some 378 households with a total population of 2344 people.

### Study design and population surveyed

Our cross-sectional study was carried out between October 2011 and August 2012 on Done Khon and Done Som islands. These study sites were selected based on a three-stage random sampling. First, we randomly selected two islands out of 10 known endemic islands for the targeted diseases. For each island, 323 study participants were required based on our sample calculation using a formula of simple random sampling, e.g., Z_1- α/2_^2^ × p(1-p)/d^2^ with a 30% proportion and 5% precision. Based on previous experiences, about 40% of all study participants (129 persons) failed to submit complete stool samples when they were asked to submit multiple stool samples (i.e., at least two). With this in mind, at least 904 study participants from both islands were required for this study. Second, two villages were selected on each island. Finally, about 30 households in each village were randomly selected to meet the required sample size. All members of the selected households, aged 2 years and older and available on the survey day, were invited to participate in the study.

Potential animal reservoir hosts, i.e., dogs, cats, pigs and buffaloes, from selected households were also enrolled and examined for helminth infections. Due to the small numbers of these animals in the study villages (0.4 animals per household; from village record), we examined all of those present during the survey. Village health volunteers helped to identify the domestic animals and conduct follow-up examinations.

We collected intermediate hosts for *O. viverrini* (*Bithynia* spp. snails and *Cyprinoid* fish) and for *S. mekongi* (*Neotricula aperta* snails) from selected sites in the study villages and examined them for infection (Fig. 1b).

Snails of the genus *Bithynia* spp. were collected with a scoop [38] from water bodies near the study villages (e.g., ponds, canals, and rice fields). From each water body, 5–10 sites with an area of 1 × 1 m were identified as collecting points. All *Bithynia* snails collected from each site were counted, recorded and examined separately. *Cyprinoid* freshwater fish were captured from the same selected water bodies as well as from the Mekong using a fishing net. Each captured fish was measured for length and weight and were examined at the field station for the presence of *O. viverrini* metacercariae.

*N. aperta* snails [39] live in the rocky area of the Mekong River. We identified 10 sites along the Mekong River, where water was frequently used by study villagers for their daily needs. Submerged stones were dredged and snails were hand-picked from them [38]. At each site, *N. aperta* snails were collected for 20 min by five malacologists. All collected snails were counted, placed in a plastic bag and carried to the field station for examination.

### Field procedures and laboratory examinations

In each village, a house, school or temple was identified as a field study station. Two questionnaires were administered to all participating households. A household questionnaire was administered to the heads of households for collecting data on household characteristics (e.g., building type, toilette and water supply), asset ownership (e.g., farm engine, boat, car, motorbike, electricity, television, bicycle, telephone and agriculture land) and animal ownership (e.g., buffalo, cow, goat and pig). An individual questionnaire was used to interview all household members to collect demographic data (e.g., age, sex, educational attainment and professional activities and behavioural risks (e.g., food consumption habits, water contact, animal raising and personal hygiene). Parents or legal guardians answered for children under 10 years of age.

Eligible study participants were invited to submit two stool samples over consecutive days for parasitological analysis. The first stool container (pre-labelled with participant’s name, unique identity number, age and date of collection) was handed to the study participants on the registration day, along with a detailed explanation of stool collection. The second empty container was handed out after study participants returned the first filled container.

Two Kato-Katz (KK) thick smears [40] were prepared from each stool sample (i.e. four smears per person) and examined under light microscopes by an experienced technician within 1 h of sample preparation. Eggs were counted and recorded for each helminth species separately.

We collected faecal samples from potential domestic reservoir animals owed by study households, namely cats, dogs, pigs and water buffaloes. To collect fresh faecal samples [41] from small animals (cats, dogs and pigs), rectal enemas were performed using Sodium Chloride (NaCl) solution and petroleum jelly lubricant. Faecal samples from water buffaloes were collected by rectal swab. All faecal samples were immediately preserved in a 10% formalin solution and transported to the National Institute of Public Health (NIOPH), Vientiane, for processing using the formalin ether concentration technique (FECT) [42].

*Bithynia* spp. and *N. aperta* snails were examined for the presence of cercariae infection using the shedding test, previously described by *Sri-Aroon* and colleagues [43]. In summary, the fresh water snails were put into a transparent plastic container filled with Mekong water and exposed to artificial light. After 2 h, the container was examined under a stereoscope for the presence of cercariae. The infected snails were identified, counted and recorded separately.

The species identification of captured *Cyprinoid* fish was performed based on guidelines available at FishBase website [44, 45]. Fish digestion was performed using the pepsin enzyme digestion technique [25]. The residue was examined for the presence of *O. viverrini* metacercariae. The metacercariae were counted and recorded for each infected fish.

### Data management and analysis

Information from questionnaires and data forms were double entered into EpiData, version 3.1 (EpiData Association; Odense, Denmark) and validated for their correctness and completeness. Statistical analyses were performed with STATA, version 13.1 (StataCorp., College Station, USA). Only study participants with at least two KK thick smear examinations and with complete questionnaires were retained in the final analysis. Participants were stratified into five age groups: (i) ≤ 9 years, (ii) 10–16 years, (iii) 17–36 years, (iv) 37–50 years, and (v) ≥ 51 years. Socioeconomic status (SES) of the household was calculated using an asset-based method. Indicator data were defined by principal component analysis (PCA). The procedure is widely used and details can be found elsewhere [5, 46, 47]. SES conditions in the household were categorized into one of five wealth quintiles, namely (i) most poor, (ii) very poor, (iii) poor, (iv) less poor, and (v) least poor according to their cumulative standardized asset scores. Details of this widely used approach have been presented elsewhere [5].

The intensity of helminth egg counts was expressed as eggs per gram of stool (EPG) obtained from Kato-Katz examination. Based on WHO recommendations, infection intensity was classified as light ﻿(*O. viverrini*: 1-999 EPG; *S. mekongi*: 1-100 EPG; hookworm: 1-1999 EPG; *T. trichiura*: 1-999 EPG; *A. lumbricoides*: 1-4999﻿ EPG), moderate ﻿﻿(*O. viverrini*: 1000-9999 EPG; *S. mekongi*: 101-400 EPG; hookworm: 2000-3999 EPG; *T. trichiura*: 1000-9999 EPG; *A. lumbricoides*: 5000-49,999﻿ EPG), and heavy (*O. viverrini*: 1-999 EPG; *S. mekongi*: 1-100 EPG; hookworm: 1-1999 EPG; *T. trichiura*: 1-999 EPG; *A. lumbricoides*: 1-4999 EPG), respectively [25, 31, 48].

Prevalence of parasitic infections was determined and stratified by age, sex and study area (Done Khon versus Done Som). Chi-square test was used to examine the association among categorical variables. The geometric mean for helminth egg counts was calculated for infected individuals. Univariate random-effects logistic regression analysis was used to associate *O. viverrini* and *S. mekongi* infections (outcome) with potential risk factors (predictors). The crude odds ratio (c*OR*), 95% confidence interval (95% *CI*) and *P-*value were calculated. Explanatory variables with a *P-*value of <15% were included in the stepwise multivariate random-effects logistic regression model. Adjusted odds ratio (a*OR*) was calculated. Smoothed age distribution of *O. viverrini*, *S. mekongi*, hookworm and *T. trichiura* infections by gender was established. Statistical significance was defined as yielding a *P-*value smaller than 0.05.

## Results

### Characteristics of the study participants

**Fig. 2:**
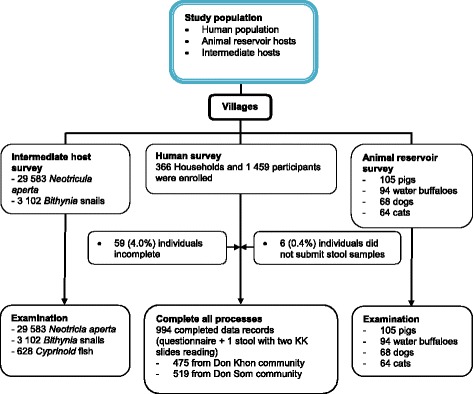
Study diagram

A total of 994 study participants were included in this final analysis (Fig. 2). Of these, 475 (47.8%) were from Done Khon and 519 (52.2%) from Done Som. There were slightly more female than male participants (51.8% vs 48.2%). Age ranged from 2 to 88 years (median age 29.8 years). The schooling rates did not differ between the two study islands. Subsistent rice farming and fishing were the main professional activities (60.0%). Less than half of the study participants reported having access to a latrine at home (Done Khon 49.7%, Done Som 38.9%). People living in Done Som had a lower socioeconomic status than in Done Khon (Most poor, 25.8% vs 16.4%, respectively). The sociodemographic characteristics of study participants are summarized in Table 1.

**Table 1:** Socio-demographic characteristics of study participants from two study islands (Done Khon and Done Som, Khong District (*n* = 994)

Characteristics	Overall *n* (%)	Study area		*x* ^*2*^	*P-*value^a^
		Done Khon, *n* (%)	Done Som, *n* (%)		
Age (years)					
Mean (range)	29.8 (2–88)	30.0 (2–87)	29.6 (2–88)		
Age group					
≤ 9	216 (21.7)	99 (20.8)	117 (22.5)		
10–16	185 (18.6)	91 (19.2)	94 (18.1)		
17–36	204 (20.5)	102 (21.5)	102 (19.7)		
37–50	203 (20.4)	88 (18.5)	115 (22.2)		
≥ 51	186 (18.7)	95 (20.0)	91 (17.5)	3.3	0.511
Sex					
Male	479 (48.2)	212 (44.6)	267 (51.5)		
Female	515 (51.8)	263 (55.4)	252 (48.6)	4.6	0.032
Educational level					
Pre-schooler	108 (10.9)	52 (10.9)	56 (10.8)		
Illiteracy	97 (9.8)	59 (12.4)	38 (7.3)		
Primary school	538 (54.1)	237 (49.9)	301 (58.0)		
High school/above	251 (25.3)	127 (26.7)	124 (23.9)	10.4	0.015
Occupation					
Preschool child	108 (10.9)	52 (11.0)	56 (10.8)		
Student	290 (29.1)	137 (28.8)	153 (29.5)		
Farmer and fisher	596 (60.0)	286 (60.2)	310 (59.7)	0.05	0.975
Socioeconomic status					
Least poor	195 (19.6)	126 (26.5)	69 (13.3)		
Less poor	203 (20.4)	73 (15.4)	130 (25.1)		
Poor	192 (19.3)	107 (22.5)	85 (16.4)		
Very poor	192 (19.3)	91 (19.2)	101 (19.5)		
Most poor	212 (21.3)	78 (16.4)	134 (25.8)	48.6	<0.001
Latrine available					
No	556 (55.9)	239 (50.3)	317 (61.1)		
Yes	438 (44.1)	236 (49.7)	202 (38.9)	11.7	0.001
Opened defecation this year					
No	484 (48.7)	256 (53.9)	228 (43.9)		
Yes	510 (51.3)	219 (46.1)	291 (56.1)	9.9	0.002

*P-*value^a^: the comparison between Done Khone and Done Som Island

### Helminth infections in humans

Helminth infections were very frequent on the two islands. *O. viverrini*, hookworm, *S. mekongi*, and *T. trichiura* were found in 60.7%, 44.1%, 22.2% and 4.1% of the participants, respectively. Very few participants were infected with *A. lumbricoides* (0.6%) and *Taenia* spp. (0.1%). The prevalence of *O. viverrini* was almost two-times higher in Done Som compared to Done Khon (77.3% vs. 42.5%*, P* < 0.001). *S. mekongi* prevalence was similar on both islands (*P* = 0.329). Multi-parasitism was diagnosed in 40.5% of the study participants. Details of the helminth infections are given in Table 2.

**Table 2:** Prevalence of *Opisthorchis viverrini*, *Schistosoma mekongi*, soil-transmitted helminth and other intestinal helminth infections among study participants from two islands (Done Khon and Done Som) of Khong District (*n* = 994)

Parasites	Positive, *n* (%) (*n* = 994)	Done Khon, *n* (%) (*n* = 475)	Done Som, *n* (%) (*n* = 519)	*x* ^*2*^	*P-*value^a^
Trematodes					
*Opisthorchis viverrini*	603 (60.7)	202 (42.5)	401 (77.3)	125.4	<0.001
*Schistosoma mekongi*	221 (22.2)	112 (23.6)	109 (21.0)	0.9	0.329
Soil-transmitted helminth					
Hookworm	438 (44.1)	196 (41.3)	242 (46.6)	2.9	0.090
*Trichuris trichiura*	41 (4.1)	21 (4.4)	20 (3.9)	0.2	0.653
*Ascaris lumbricoides*	6 (0.6)	6 (1.3)	0	6.6	0.010
Cestodes					
*Taenia*spp.	1 (0.1)	1 (0.2)	0	1.1	0.296
Multiparasitism					
No infection	202 (20.3)	127 (26.7)	75 (14.5)		
Single species	379 (38.1)	197 (41.5)	182 (35.1)		
Multiple species	413 (40.5)	151 (31.8)	261 (40.5)	43.9	<0.001

*P-*value^a^: the comparison between Done Khone and Done Som Island

**Fig. 3:**
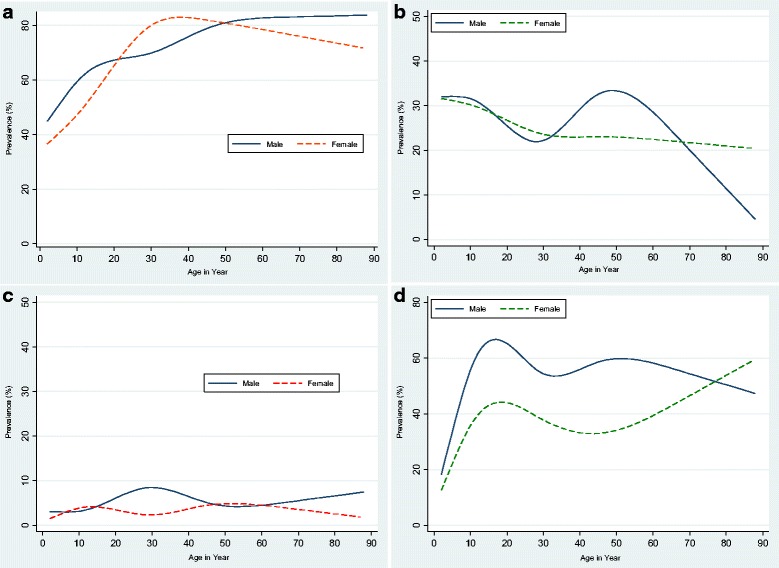
Age distribution of major helminth infections by gender on Done Khon and Done Som islands. The figures represent the smoothed age distribution of male (*solid line*) and female (*dotted line*) study participants for an infection with (**a**): *Opisthorchis viverrini*, (**b**): hookworm, (**c**): *Trichuris trichiura* and (**d**): *Schistosoma mekongi*

Figure 3 displays the smoothed age prevalence of helminth infections by gender. *O. viverrini* infection appears to be acquired at a young age, with prevalence increasing gradually (Fig. 3a). Hookworm infection is acquired at a very young age. For males, the prevalence peaked among adolescents aged 10–20 years and plateaued among older age groups. For females, prevalence peaked between 10 and 20 years old and again after 50 years old (Fig. 3b). For males, two prevalence peaks were observed; the first among children under 10 years old and the second among adults between 40 and 50 years old. For females, only one peak was seen among children under 10 years old. *T. trichiura* prevalence was distributed similarly among males and females independent of age (Fig. 3c). *S. mekongi* prevalence was differently distributed among males and females (Fig. 3d).

Human helminth infection intensities are summarized in Table 3. Most helminth infections were categorized as light infections. Nevertheless, *O. viverrini, S. mekongi* and hookworm accounted for infections of heavy intensity in some cases (4.2%, 3.6% and 1.8%, respectively).

**Table 3:** Infection intensity of *Opisthorchis viverrini, Schistosoma mekongi* and soil-transmitted helminths among study participants from two islands (Done Khon and Done Som) of Khong District (*n* = 994)

Infections	Light			Moderate			Heavy		
	Overall	Done Khon	Done Som	Overall	Done Khon	Done Som	Overall	Done Khon	Done Som
	*n* (%)	*n* (%)	*n* (%)	*n* (%)	*n* (%)	*n* (%)	*n* (%)	*n* (%)	*n* (%)
*Opisthorchis viverrini*	409 (67.8)	174 (86.1)	235 (58.6)	169 (28.0)	27 (13.4)	142 (35.4)	25 (4.2)	1 (0.5)	24 (6.0)
*Schistosoma mekongi*	187 (84.6)	100 (89.3)	87 (79.8)	26 (11.8)	10 (8.9)	16 (14.7)	8 (3.6)	2 (1.8)	6 (5.5)
Hookworm	420 (95.9)	191 (97.5)	229 (94.6)	10 (2.3)	2 (1.0)	8 (3.3)	8 (1.8)	3 (1.5)	5 (2.1)
*Trichuris trichiura*	41 (97.6)	22 (100.0)	19 (95.0)	1 (2.4)	0	1 (5.0)	0	0	0
*Ascaris lumbricoides*	5 (83.3)	5 (83.3)	0	1 (16.7)	1 (16.7)	0	0	0	0

### Prevalence of helminth infections in animal reservoirs and intermediate hosts

Table 4 summarizes the results of infections in animals, snails and *Cyprinoid* fish. Analysis of animal faeces showed that overall prevalence of *O. viverrini* infection in cats, dogs and pigs was 53.1%, 25.0% and 0.9%, respectively, while only dogs (14.7%) were found to be infected with *S. mekongi*. Examination of intermediate host snails for *O. viverrini* (*Bithynia* spp.,) and for *S. mekongi* (*N. aperta*) detected infection rates of 0.3% and 0.01%, respectively (Table 4). A similar rate of *O. viverrini* infection was found in *Bithynia* spp. from Done Khon and Done Som (0.1% vs. 0.5%, *P* = 0.045), while only the *N. aperta* snails from Done Khon (0.02%) were found to be infected with *S. mekongi.*

**Table 4:** Prevalence of *Opisthorchis viverrini *and ﻿*Schistosoma mekongi* infections in animals on Done Khon and Done Som islands

Infections	No. exam	Overall, *n* (%)	Done Khon, *n* (%)		Done Som, *n* (%)		*x* ^*2*^	*P-*value^a^
			No. exam	No. positive	No. exam	No. positive		
*Opisthorchis viverrini*								
Dog	68	17 (25.0)	44	10 (22.7)	24	7 (29.2)	0.34	0.558
Cat	64	34 (53.1)	25	15 (60.0)	39	19 (48.7)	0.78	0.378
Pig	105	1 (0.9)	43	0	62	1 (1.6)	0.70	0.403
Water buffalo	94	0	32	0	62	0	na	na
Intermediate snails								
*Bithynia* spp.	3102	9 (0.3)	1719	2 (0.1)	1383	7 (0.5)	4.03	0.045
Minute intestinal fluke (MIF)								
Dog	68	3 (4.4)	44	3 (6.8)	24	0	1.71	0.191
Cat	64	18 (28.1)	25	5 (20.0)	39	13 (33.3)	1.33	0.247
Large trematode eggs								
Water buffaloes	94	18 (19.1)	32	9 (28.1)	62	9 (14.5)	2.52	0.112
Pig	105	4 (3.8)	43	2 (4.6)	62	2 (3.2)	0.14	0.708
*Schistosoma mekongi*								
Dog	68	10 (14.7)	44	7 (16.0)	24	3 (13.0)	0.14	0.704
Cat	64	0	25	0	39	0	na	
Pig	105	0	43	0	62	0	na	
Water buffalo	94	0	32	0	62	0	na	
Intermediate snails								
*Neotricula aperta*	29,583	4 (0.01)	16,342	4 (0.02)	13,241	0	3.24	0.072

*P-*value^a^: the comparison between Done Khone and Done Som Island

*na* Not applicable

Table 5 displays the prevalence of *O. viverrini* infection in the *Cyprinoid* fish collected from habitats in Done Khon and Done Som islands. In total, 628 fish representing 21 species were digested and examined. Of these, 622 represented 19 species of *Cyprinoid* fish, five fish were from the Osphronemidiae family and one fish from the Anabantidae family. Only Cyprinoidae fish species were infected with *O. viverrini*, with an overall prevalence of 26.9% and an average of 228.7 metacercariae per fish. The highest infection intensity was seen in *Cyclocheilichthys apogon,* with an average of 168.7 metacercariae per infected fish. Only one fish of the *Anabas testudineus* from Anabantidae family was examined. It was found positive for minute intestinal fluke metacercariae.

**Table 5:** Prevalence of *Opisthorchis viverrini* and minute intestinal flukes (MIF) metacercariae in cyprinoid fish from Done Khon and Done Som islands

Scientific name Species	Lao name	No. exam.\	No. of fish infected *O. viverrini* positive (%)	No. of *O. viverrini* metacercariae Mean, SD (range)	No. of fish infected MIF positive (%)	No. of MIF metacercariae Mean, SD (range)	Weight (gram) Mean, SD (range)
*Morulius chrysophekadion*	Pa phea	1	1 (100.0)	2.0, na	0	0	138 (na)
*Hampala dispa*	Pa soud	101	88 (87.1)	112.1, ± 188.0 (3–1468)	9 (8.9)	8.6, ± 10.6 (1–48)	11.4, ± 11.1 (1.9–66.4)
*Cyclocheilichthys apogon*	Pa dok-ngew	21	18 (85.7)	168.7, ± 283.9 (2–984)	5 (23.8)	6.4, ± 4.4 (2–12)	7.1, ± 4.6 (1.5–20.1)
*Puntius brevis*	Pa khao-mon	100	40 (40.0)	120.2, ± 322.2 (1–1940)	22 (22.2)	10.7, ± 10.9 (1–48)	8.5, ± 9.3 (1.1–39.1)
*Henicorhynchus lineatus*	Pa soi	14	3 (21.4)	31, ± 37.3 (7–74)	0	0	11.8, ± 5.3 (3.7–23)
*Barbonymus gonionotus*	Pa pak-khao	16	2 (13.0)	10, ± 7.1 (5–15)	0	0	38.2, ± 25.5 (3.9–84.9)
*Barbonymus altus*	Pa wien-fai	17	2 (11.8)	2.5, ± 0.7 (2–3)	0	0	21.5, ± 7.5 (2.9–36.1)
*Poropuntius deauratus*	Pa chad	163	10 (6.1)	21.6, ± 43.6 (1–142)	16 (9.8)	9.3, ± 12.6 (1–42)	14.3, ± 23.4 (1.4–148.6)
*Puntioplites falcifer*	Pa sa-khang	34	2 (6.0)	6.0, ± 5.7 (2–10)	1 (2.9)	2.0, na	19.2, ± 8.8 (3.3–38.7)
*Scaphognathops bandanensis*	Pa pieng	40	2 (5.0)	1.5, ± 0.7 (1–2)	2 (5.0)	7.5, ± 7.8 (2–13)	33.1, ± 14.9 (6.2–68.7)
*Albulichthys albuloides*	Pa ta-sai	69	1 (1.5)	1, na	22 (31.9)	19.7, ± 34.9 (1–132)	14.4, ± 5.3 (4.9–27.9)
*Opsarius koratensis*	Pa sew-oua	16	0	0	1 (6.3)	2.0, na	6.3, ± 5.3 (1.9–14.6)
*Paralaubuca typus*	Pa tab	5	0	0	1 (20.0)	2.0, na	8.5, ± 5.1 (5.1–17.3)
*Mystacoleucus atridorsalis*	Pa lang-khon	9	0	0	0	0	9.1, ± 14.1 (1.4–36.9)
*Cyclocheilichthys enoplus*	Pa choox	5	0	0	0	0	30.7, ± 11.9 (18.6–48.6)
*Luciosoma bleekeri*	Pa mak-vai	4	0	0	0	0	26.1, ± 3.9 (23.7–31.9)
*Osteochilus melanopleurus*	Pa nok-khao	3	0	0	0	0	8.8, ± 9.7 (2.8–20)
*Raiamas guttatus*	Pa sa-nak	3	0	0	0	0	38.6, ± 29.8 (10–69.4)
*Probarbus labeamajor*	Pa oearn	1	0	0	0	0	45.9 (na)
*Trichogaster trichopterus* ^*a*^	Pa ka-deuth	5	0	0	0	0	4.6, ± 2.4 (2.3–7.8)
*Anabas testudineus* ^*b*^	Pa kheng	1	0	0	1 (100)	3.0, na	9.7 (na)
Total		628	169 (26.9)	106.9 ± 228.7 (1–1940)	12.7	11.9, ±20.7 (1–132)	15.0, ±17.4 (1.1–148.6)

Belongs to the

^a^Osphronemidae and

^b^Anabantidae family, *NA* Not appropriate, *SD* standard deviation, *No* Numb

### Risk factor analysis for *O. viverrini* and *S. mekongi* infections in human

Table 6 shows the association between risk factors of *O. viverrini * and *S. mekongi * infections. The stepwise multivariate analysis showed that illiteracy (illiteracy vs. preschool children: a*OR* = 6.0, 95% *CI*: 3.3–11.0), *P* = 0.028) and lower socioeconomic status were associated with an increased risk of being infected with *O. viverrini* (less poor vs least poor: a*OR* = 3.1, 95% *CI*: 1.7–7.5, *P* = 0.013), while school children in the age group 10–16 years (a*OR* = 0.1, 95% *CI*: < 0.1–0.4, *P =* 0.003) and those with a latrine at home (a*OR* = 0.2, 95% *CI*: 0.1–0.4), *P* = 0.001) were more likely to be protected against the infection. Furthermore, having household dogs and cats that eat raw fish was significantly and positively associated with *O. viverrini* infection of the household members (a*OR* = 1.9, 95% *CI*: 1.2–3.1, *P* = 0.007). The age group was the only factor significantly associated with *S. mekongi* infection. Children in the age group ≤9 years old were significantly exposed to this infection compared to older age groups (age group 10–16: a*OR* = 0.5, 95% *CI*: 0.2–0.9, *P* = 0.047, age group 17–36: a*OR* = 0.2, 95% *CI*: < 0.1–0.8, *P =* 0.022; age group 37–50: a*OR* = 0.2, 95% *CI*: < 0.1–0.8, *P =* 0.021 and age group ≥51: a*OR* = 0.2, 95% *CI*: < 0.1–0.8, *P =* 0.024). The model revealed that age group (10–16 year: a*OR* = 1.7, 95% *CI:* 1.1–2.7, *P* = 0.015), educational level (illiteracy: a*OR* = 7.4, 95% *CI*: 3.2–17.3, *P* < 0.001, and primary school: a*OR* = 4.8, 95% *CI*: 2.0–11.3, *P* < 0.001) and raising pigs at home (a*OR* = 1.3, 95% *CI*: 1.1–1.7, *P* = 0.047) were significant risk factors for STH infection, while being a women (a*OR* = 0.4, 95% *CI*: 0.3–0.6, *P* < 0.001) or having a latrine at home (a*OR* = 0.6, 95% *CI*: 0.4–0.8, *P* < 0.001) were protective factors.

**Table 6:** Stepwise multivariate logistic regression (backward elimination) analyses the association between underlying risk factors and *O. viverrini*, *S. mekongi* and STH infections among study participants on both islands (Done Khon and Done Som islands (*n* = 994)

Characteristics	*O. viverrini*				*S. mekongi*				Soil-transmitted helminth			
	Crude *OR* (95% *CI*)	*P-value*	Adjusted *OR* (95% *CI*)	*P*-value	Crude *OR* (95% *CI*)	*P*-value	Adjusted *OR* (95% *CI*)	*P*-value	Crude *OR* (95% *CI*)	*P*-value	Adjusted *OR* (95% *CI*)	*P*-value
≤ 9	1		1		1		1		1		1	
10–16	1.6 (1.1–2.4)	0.022	0.1 (< 0.1–0.4)	0.003	0.6 (0.4–1.1)	0.075	0.5 (0.2–0.9)	0.047	2.7 (1.8–4.0)	< 0.001	1.7 (1.1–2.7)	0.015
17–36	3.3 (2.2–4.9)	< 0.001	NS	NS	0.5 (0.3–0.8)	0.005	0.2 (< 0.1–0.8)	0.022	1.9 (1.3–2.9)	0.001	NS	NS
37–50	4.3 (2.8–6.4)	< 0.001	NS	NS	0.5 (0.4–0.9)	0.006	0.2 (< 0.1–0.8)	0.021	2.2 (1.5–3.2)	< 0.001	NS	NS
≥ 51	4.2 (2.7–6.4)	< 0.001	NS	NS	0.5 (0.4–0.9)	0.011	0.2 (< 0.1–0.8)	0.024	2.4 (1.6–3.6)	< 0.001	NS	NS
Sex												
Male/Female	1/1.1 (0.8–1.5)	0.579	NA	NA	1/0.8 (0.6–1.2)	0.401	NA	NA	1/0.5 (0.4–0.6)	< 0.001	1/0.4 (0.3–0.6)	< 0.001
Educational level												
Preschooler	1		1		1		1		1		1	
Illiteracy	6.0 (3.3–11.0)	< 0.001	9.4 (1.3–68.9)	0.028	0.6 (0.3–1.2)	0.131	NS	NS	4.0 (2.2–7.2)	< 0.001	7.4 (3.2–17.3)	< 0.001
Primary school	4.0 (2.5–6.2)	< 0.001	NS	NS	0.7 (0.3–1.2)	0.140	NS	NS	2.9 (1.8–4.6)	< 0.001	4.8 (2.0–11.3)	< 0.001
High school/above	2.7 (1.7–4.3)	< 0.001	NS	NS	0.6 (0.4–1.1)	0.101	NS	NS	2.8 (1.7–4.6)	< 0.001	NS	NS
Occupation												
Preschool child	1		1		1		1		1		1	
Student	1.2 (0.8–1.8)	0.377	NA	NA	1.5 (1.0–1.9)	0.034	NS	NS	1.9 (1.2–2.8)	0.003	NS	NS
Farmer	3.1 (2.1–4.6)	< 0.001	NS	NS	2.0 (1.0–2.6)	0.017	NS	NS	1.8 (1.3–2.7)	0.002	NS	NS
Socio - economic status												
Least poor	1		1		1		1		1		1	
Less poor	2.4 (1.3–4.7)	0.007	6.5 (1.2–37.5)	0.037	1.5 (0.9–2.3)	0.321	NA	NA	1.5 (1.0–2.2)	0.041	NS	NS
Poor	2.2 (1.1–4.2)	0.018	NS	NS	1.9 (1.1–2.9)	0.082	NS	NS	1.2 (0.8–1.8)	0.340	NA	NA
Very poor	1.5 (0.8–2.9)	0.213	NS	NS	0.9 (0.5–1.6)	0.816	NA	NA	1.2 (0.8–1.8)	0.395	NA	NA
Most poor	3.7 (2.0–7.0)	< 0.001	NS	NS	0.9 (0.6–1.6)	0.753	NA	NA	1.3 (0.9–1.9)	0.209	NA	NA
Latrine available												
No/Yes	1/0.4 (0.3–0.7)	< 0.001	1/0.2 (0.1–0.4)	0.001	1/0.8 (0.6–1.1)	0.148	NA	NA	1/0.6 (0.5–0.8)	< 0.001	1/0.6 (0.4–0.8)	< 0.001
Has ever heard about diseases												
No/Yes	1/1.5 (0.9–2.3)	0.090	NS	NS	1/0.8 (0.6–1.1)	0.154	NA	NA	NA	NA	NA	NA
Known about transmission route									NA	NA	NA	NA
No/Yes	1/1.8 (1.0–3.2)	0.041	NS	NS	1/0.8 (0.5–1.5)	0.660	NA	NA	NA	NA	NA	NA
Open defecation this year												
No/Yes	1/1.8 (1.4–2.3)	< 0.001	NS	NS	1/1.1 (0.8–1.5)	0.482	NA	NA	1/1.6 (1.2–2.1)	< 0.001	NS	NS
Water contact for fishing/farming												
No/Yes	1/1.5 (1.0–2.1)	0.038	NS	NS	1/0.9 (0.6–1.4)	0.730	NA	NA	1/1.6 (1.2–2.3)	< 0.005	NS	NS
Eating raw/undercooked fish												
No/Yes	1/4.3 (2.6–6.9)	< 0.001	NS	NS	1/0.6 (0.4–0.8)	0.004	NS	NS	1/0.8(0.5–3.2)	0.872	NS	NS
Raising cats at home												
No/Yes	1/1.0 (0.8–1.3)	0.959	NA	NA	1/0.8 (0.6–1.2)	0.542	NA	NA	1/1.2 (0.9–1.6)	0.094	NS	NS
Raising dogs at home												
No/Yes	1/0.9 (0.7–1.2)	0.397	NA	NA	1/0.7 (0.4–1.4)	0.343	NA	NA	1/1.2 (0.9–1.5)	0.132	NS	NS
Raising pigs at home												
No/Yes	1.1 (0.9–1.5)	0.398	NA	NA	1.2 (0.9–1.6)	0.336	NA	NA	1/1.2 (0.9–1.6)	0.132	1/1.3 (1.1–1.7)	0.047
Raising buffaloes at home												
No/Yes	1/1.1 (0.9–1.5)	0.394	NA	NA	1/1.3 (0.9–1.8)	0.133	NS	NS	1/1.1 (0.8–1.2)	0.845	NA	NA
Observed dog/cat eat raw/undercooked fish												
No/Yes	1/1.3 (0.9–1.6)	0.059	1/1.9 (1.2–3.1)	0.007	NA	NA	NA	NA	1/1.3 (0.8–1.7)	0.169	NA	NA

*NA* Not appropriate for analysis (all variables with *P*-value ≥15% and are removed by model), *NS* Not significant (all variables with *P*-value <15%, but are not significant after adjusted analysis

## Discussion

The Khong District, with its dozens of islands in the Mekong, has a distinct ecological setting (Fig. 1). Human settlements line the island shores, while the rest of the island is used for agricultural activities, particularly rice farming. The Mekong River as well as the diverse water bodies on the islands represent a rich ecosystem for fish and mollusc populations. On two Mekong islands, highly endemic for multiple species of helminth infections, we studied the transmission of *O. viverrini, S. mekongi* and STH using an ecohealth approach [32, 37] to better assess the relation of human infection status to environmentally present reservoir and intermediate hosts. Heavy infections and multi-parasitism were prevalent among the human population and age-gender distributions revealed parasite-specific patterns. Examination of potential animal reservoir hosts from the study participants’ households (cats, dogs, pigs and buffaloes) yielded ten different helminth species, with many of them having zoonotic capacity. Infection rates of intermediate snail hosts *Bithynia* sp. and *N. aperta* were low but reflect on-going transmission. In addition, infection rates of locally caught cyprinoid fish with *O. viverrini* and minute intestinal fluke (MIF) metacercariae were very high, pointing to a high risk of infection when they are consumed raw or undercooked.

In this study, we document high infection rates of *O. viverrini, S. mekongi* and selected species of STH, namely hookworm infections. The high infection rates are a surprise given that MDA campaigns were conducted annually between 2008 and 2013 [26], in which praziquantel (40 mg/kg BW single dose) and albendazole (400 mg single dose) were provided to the entire population (older than 4 years). In addition, biannual deworming (with mebendazole) takes place in all Lao primary schools [27]. Local health authorities confirmed that all Mekong islands were targeted, but we could not find coherent information on the number of treatment rounds conducted on our study islands. Nevertheless, our results indicate that the impact of the intervention is insufficient.

The Ministry of Health’s objective is to eliminate *S. mekongi* as a public health problem in Lao PDR by 2016. On our study islands, *S. mekongi* cannot be considered eliminated given the high infection rates. Our data indicate that *S. mekongi* infection in dogs may fuel the transmission by constantly infecting *Neotricula* populations in the Mekong. Of similar importance are cats and dogs for the transmission of *O. viverrini*. Hence, animal reservoirs in households should also be a target of integrated parasite control on the Mekong islands, and throughout Lao PDR.

Several factors might account for the persisting high *O. viverrini* infection rates among humans on the Mekong islands. One such factor is the high infection prevalence among cyprinoid fish. More than 80 species of the Cyprinidae family and at least 13 species of other families can serve as a secondary intermediate host [25]. In our study, *O. viverrini* metacercariae were identified in 11 cyprinoid fish species, while some had particularly high *O. viverrini* metacercariae infection rates, e.g. in 87.1% of *Hampala dispa*. All the cyprinoid species in which we detected an infection are known to be good *O. viverrini* transmitting species [49–52]. They were identified in all water bodies examined in this study. Fish are mostly likely infected while small and living in rice fields, canals and ponds. The metacercariae remain alive as the fish grow and move into the Mekong.

Cyprinoid fish accumulate the metacercariae over a long time. Low infection rates in *Bithynia* snails may be sufficient for transmission [53]. We found a low infection rate of 0.3% in *Bithynia* sp. snails. Other studies have detected infection rates between 0.3–8.3% [54]. But infection rates may vary considerably, depending on sampling locality and season [54, 55]. It is important to note that even low infection prevalence rates are sufficient for maintaining transmission.

We observed low *S. mekongi* infection rates in *N. aperta* (0.02%) compared to other reports*.* The presence of infected molluscs gives evidence that *S. mekongi* transmission is currently on-going. Therefore, abandoning control activities would inevitably lead to an increase in infection rates among humans. There are many more *S. mekongi* endemic Mekong islands, which might display a different *N. aperta* population distribution and infection pattern [9, 10].

A major finding from our study is the dramatically high helminth infection rates among domestic cats, dogs, pigs and buffaloes. Ten different parasite species were detected in these animal hosts residing in the households of our study participants. By using FECT, we could distinguish *O. viverrini* eggs in dogs and cats from other small trematode eggs. Our results showed higher rates than Aunpromma et al. (2012) found in neighbouring Thailand, where 0.37% and 35.5% of the dogs and cats were infected, respectively [56]. The infection rate among dogs, in particular, was 20 times higher than that found in the study of Aunpromma et al. (2012). Through observation and from interviewing animal owners in both communities, it appears that most of the dogs and cats were free-roaming and usually accompanied their owners to the rice field where they caught and ate fish directly from the canals or rice fields. Moreover, raw and undercooked fish were often fed to these animals. These phenomena, in combination with the high infection rates of dogs and cats, likely maintain the transmission of *O. viverrini* and other fish-borne trematode infections in the communities.

Only dogs were diagnosed with *S. mekongi* in this study, which is consistent with other study findings [9, 22]. We did not find any *S. mekongi* eggs in pigs or water buffaloes, though both animals were found to be infected in earlier investigations [57]. However, they are not of importance for transmission on our study islands. On other Mekong islands where these animals are more free-roaming, their infection status could be higher and, thus, their contribution to transmission of greater importance.

The results of our risk factor analysis for *O. viverrini* infection differed from many previous studies [5, 30, 49]. More than half of our risk factors dropped out after multivariate analysis, whereas the initial univariate analysis showed significant associations between infection and age group, occupation, socioeconomic status, latrine availability, history of open defecation this year, and eating raw and/or undercooked fish (Table 6). The association between *O. viverrini* and socioeconomic status was not clear for our study population. The study area was geographically very small. Therefore, the variation in socioeconomic status and living conditions might not have varied enough to results in risk differentiation. Furthermore, control activities such as the annual treatments between 2008 and 2013, have had an impact on infection status, which in turn might have blurred important associations. For example, eating raw/undercooked fish was not significantly associated with *O. viverrini* infection, although deeply rooted habits of eating raw or improperly cooked fish is a well-known factor in sustaining helminth infections in humans and difficult to control [30, 53, 58].

In our multivariable analysis, we did not find any association between *S. mekongi* infection and risk factors, except for age. Children under 9 years old had a higher risk of infection than older study participants. This result is likely due to MDA over the years having reduced infection rates among older villagers. Therefore, controls targeting lower age groups could further contribute to eliminating *S. mekongi* on the Mekong islands.

Our study suffers from some limitations. Our diagnostic procedure most likely underestimated the true infection burden. Although examining a duplicate Kato-Katz thick smear per faecal sample has a considerably higher sensitivity than a single smear, the egg detection rate remains far below that of a multiple stool sample diagnostic procedure [5, 18]. Furthermore, the Kato-Katz technique cannot differentiate small trematode eggs [59]. It is therefore possible that some of the infections in humans were counted as *O. viverrini* infections instead of MIF.

## Conclusions

We conclude that human intestinal helminth infections, namely *O. viverrini, S. mekongi* and hookworms are still highly endemic on the Mekong islands in Khong District. The low prevalence of *O. viverrini* and *S. mekongi* infection in intermediate snail hosts point at on-going transmission. Animal reservoir hosts, particularly cats and dogs, have high *O. viverrini* infection rates, while only dogs are infected with *S. mekongi*. An appropriate integrated control approach involving interventions targeting human behaviour, animal reservoirs, and environmental modification might improve the effectiveness of interventions and lead to the elimination of infections.

## Additional file

Additional file 1: Multilingual abstract in the five official working languages of the United Nations. (PDF 975 kb)

## Abbreviations

95% *CI*: 95% confidence interval
*A. duodenale*: *Ancylostoma duodenale*
*A. lumbricoides*: *Ascaris lumbricoides*
a*OR*: Adjusted odds ratio
BW: Body weight
CCA: Cholangiocarcinoma
cOR: Crude odds ratio
EPG: Eggs per gram of stool
FECT: Formalin ether concentration technique
IEC: Information, education and communication
KAPP: Knowledge, attitude, practice and perception
Lao PDR: Lao People’s Democratic Republic
LMIC: Low and middle income countries
MDA: Mass drug administration
MIF: Minute intestinal flukes
*N. americanus*: *Necator americanus*
*N. aperta*: *Neotricula aperta*
NaCl: Sodium chloride
NIOPH: National Institute of Public Health
NTDs: Neglected tropical diseases
*O. viverrini*: *Opisthorchis viverrini*
PCA: Principle Component Analysis
*S. mekongi*: *Schistosoma mekongi*
*S. stercoralis*: *Strongyloides stercoralis*
STH: Soil transmitted helminth
*T. trichiura*: *Trichuris trichiura*
WHO: World Health Organization
