# Supplementary material for: Transmission of Opisthorchis viverrini, Schistosoma mekongi and soil-transmitted helminthes on the Mekong Islands, Southern Lao PDR
Source: Infect Dis Poverty. 2017 Sep 4;6:131. doi: 10.1186/s40249-017-0343-x (PMC5582398; doi:10.1186/s40249-017-0343-x)
Supplement: Additional file 1: — Multilingual abstract in the five official working languages of the United Nations. (PDF 975 kb) [file 40249_2017_343_MOESM1_ESM.pdf]

Translation of the abstract into the six official working languages of the United Nations

انتقال متأخر الخصية الزبادي والبلهارسيا الميكونجية والديدان المنقولة بالتربة في جزر ميكونج، جنوب جمهورية لاو الديمقراطية الشعبية

يوثانافانه فونجاشاك، بيتر أودرمات، كيوكا تيسايافونج، سوفانه فونزافاث، كونجساب أكافونج، سومفو ساياسون

ملخص

خلفية: انتشار متأخر الخصية الزبادي، والبلهارسيا الميكونجية والديدان المنقولة بالتربة (*STH*) لا يزال مرتفع في جمهورية لاو الديمقراطية الشعبية، على الرغم من جهود المكافحة بما في ذلك توزيع الأدوية على نطاق جماهيري، وحملات التثقيف والاتصال. وهناك حاجة إلى نهج جديدة للنهوض بمكافحة الديدان.

المنهجية: أجريت دراسة عن الصحة الإيكولوجية في جزيرتي ميكونج في جنوب لاوس. تم جمع البيانات الديموجرافية والسلوكية من خلال استبيان. تم فحص براز البشري والحيواني. تم فحص قواقع أنواع Bithynia spp. وNeotricula aperta باستخدام الفصل. تم فحص أسماك المياه العذبة باستخدام تقنية الهضم. تم استخدام تحليل الآثار العشوائية متعددة المتغيرات للعثور على عوامل الخطر المرتبطة بالتهابات الديدان الطفيلية.

النتائج الرئيسية: معدلات العدوى البشرية بأنواع متأخر الخصية الزبادي، الدودة الخطافية، 4البلهارسيا الميكونجية، الدودة السوطية، 5دود الصفر الأسطواني والشريطة كانت 60.7٪ و44.1٪ و22.2٪ و4.1٪ و0.6٪ و0.1٪ على التوالي. وكانت العدوى الشديدة الكثافة 4.2٪، 3.6٪ و1.8٪ لمتأخر الخصية الزبادي، البلهارسيا الميكونجية والدودة الخطافية، على التوالي. متأخر الخصية الزبادي والبلهارسيا الميكونجية كانت معدلات الإصابة بين الكلاب والقطط 25.0٪ و14.7٪ على التوالي. من القطط التي تم اختبارها، 53.1٪ كانت مصابة 11بمتأخر الخصية الزبادي. انتشار متأخر الخصية الزبادي والبلهارسيا الميكونجية في القواقع 0.3٪ و0.01٪، على التوالي. الانتشار الكلي للـO. viverrini كانت الإصابة في أسماك المياه العذبة 26.9٪، مع أعلى معدلات الإصابة التي تحدث في Hampala dispa (%87.1) Cyclocheilichthys apogon (%85.7) وPuntius brevis (%40). في حين أن الذين تتراوح أعمارهم بين 10-16 سنة وكان لديهم مراحيض في المنزل كانوا أقل عرضة للإصابة. وكانت الكلاب والقطط المنزلية التي تستهلك الأسماك النيئة مرتبطة ارتباطا كبيرا وإيجابيا 23بإصابة أفراد الأسرة بعدوى متأخر الخصية الزبادي بالنسبة إلى البلهارسيا الميكونجية، فإن الأطفال دون سن 9 سنوات تعرضوا بشكل كبير لهذه العدوى، مقارنة مع الفئات العمرية الأكبر سنا.

الاستنتاجات: هناك حاجة ماسة إلى تصميم وتنفيذ تدخل متكامل لمكافحة الديدان الطفيلية في جزر ميكونج جنوب جمهورية لاو الديمقراطية الشعبية. وبالنظر إلى قدرة الانتقال التي تتميز بديناميكية عالية في البلهارسيا الميكونجية، ومتأخر الخصية الزبادي والديدان المنقولة عن طريق التربة، وتعدد الطفيليات المنتشر، فإن إعطاء الأدوية على نطاق واسع سنويا أمر ضروري بجانب التعديلات البيئية والتعليم الصحي وتحسين فرص الحصول على المياه النظيفة والصرف الصحي الكافي لتعزيز السيطرة على الأمراض والمضي قدما نحو القضاء عليها.

Translated from English version into Arabic by Mahmoud Sami, through

TRANSLATORS
WITHOUT BORDERS

老挝南部湄公河岛屿上麝猫后睾吸虫（*Opisthorchis viverrini*）、湄公血吸虫（*Schistosoma mekongi*）和土源性蠕虫的传播

Youthanavanh Vonghachack, Peter Odermatt, Keoka Taisayyavong, Souphanh Phounsavath, Kongsap Akkavong, Somphou Sayasone

摘要

引言：尽管已采取了大规模化疗和健康教育措施，在老挝麝猫后睾吸虫（*Opisthorchis viverrini*）、湄公血吸虫（*Schistosoma mekongi*）和土源性蠕虫的流行率依然很高。因此，需要新的方法来推进蠕虫病的防治。

方法: 在老挝南部的两个湄公河岛屿上开展了一项生态健康研究。通过问卷收集了人口和行为数据。检查人畜粪便。对 *Bithynia* spp.和开放新拟钉螺（*Neotricula aperta*）进行脱壳检测，采用消化技术检查新鲜淡水鱼类。采用多变量随机效应模型分析与蠕虫感染相关的危险因素。
结果： 麝猫后睾吸虫、钩虫、湄公血吸虫、鞭虫、蛔虫和带绦虫的人感染率分别为 60.7％、44.1％、22.2％、4.1％、0.6％和 0.1％。麝猫后睾吸虫、湄公血吸虫、钩虫的重度感染率分别为 4.2％、3.6％和 1.8％。狗和猫的麝猫后睾吸虫、湄公血吸虫感染率分别为 25.0％和 14.7％。在所检测的猫中，53.1％感染了麝猫后睾吸虫。钉螺的麝猫后睾吸虫和湄公血吸虫感染率分别为 0.3％和 0.01％。淡水鱼的麝猫后睾吸虫总感染率为 26.9％，其中感染率最高的为 *Hampala dispa* （87.1％）、*Cyclocheilichthys apogon*（85.7％）和 *Puntius brevis*（40.0％）。文盲和社会经济较低的人群感染麝猫后睾吸虫的风险较高，而年龄在 10～16 岁且在家中拥有厕所的人群受感染的机会较小。家庭成员感染麝猫后睾吸虫与家庭中有进食生鱼的猫和狗呈显著正相关。对于湄公血吸虫，与老年人相比，9 岁以下的儿童显著暴露于感染。
结论： 本研究认为迫切需要在老挝南部湄公河群岛设计和实施一个综合的蠕虫控制干预方案。 鉴于麝猫后睾吸虫、湄公血吸虫和土源性蠕虫的高度动态传播和多重寄生的扩张，必须保证每年进行大规模化疗，同时进行环境改造、健康教育，以及提高清洁水源和卫生设施的可及性，以巩固和控制发病率并达到消除。

Translated from English version into Chinese by Xin-Yu Feng and Yin-Long Li, edited by Pin Yang

CHINESE CENTER FOR DISEASE CONTROL AND PREVENTION
NATIONAL INSTITUTE OF PARASITIC DISEASES

Transmission d'*Opisthorchis viverrini*, *Schistosoma mekongi* et d'helminthes transmis par le sol dans les îles du Mékong, au sud de la RDP Lao

Youthanavanh Vonghachack, Peter Odermatt, Keoka Taisayyavong, Souphanh Phounsavath, Kongsap Akkavong, Somphou Sayasone

Résumé
Arrière-plan: La prévalence d'*Opisthorchis viverrini,* de *Schistosoma mekongi* et d'helminthes transmis par le sol (STH) reste élevée dans la République Démocratique Populaire du Laos (RDP Lao), malgré les efforts de contrôle comprenant les campagnes de distribution massive de médicaments, d'éducation et de communication. De nouvelles approches sont nécessaires pour faire avancer la lutte contre les helminthes.
Méthodologie: Une étude sur l'écosanté a été menée sur deux îles du Mékong au sud du Laos. Les données démographiques et comportementales ont été recueillies par questionnaire. Les selles de réservoirs humains et animaux ont été examinées. Les escargots *Bithynia* spp. et *Neotricula aperta* ont été examinés à l'aide de leurs excrétions. Les poissons d'eau douce ont été examinés à l'aide d'une technique de digestion. Une analyse multivariée des effets aléatoires a été utilisée pour trouver les facteurs de risque associés aux infections par les helminthes.
Principaux résultats: Les taux d'infection humaine par *O. viverrini*, l'ankylostome, *S. mekongi, Trichuris trichiura*, *Ascaris lumbricoides* et *Taenia* spp. étaient respectivement de 60.7 %, 44.1 %, 22.2 %, 4.1 %, 0.6 % et 0.1 %. Les infections à forte intensité étaient de 4.2 %, 3.6 % et 1.8 % pour respectivement *O. viverrini*, *S. mekongi* et l'ankylostome. Les taux d'infection par *O. viverrini et S. mekongi* chez les chiens et les chats étaient respectivement de 25,0 % et 14,7 %. Parmi les chats testés, 53,1% étaient été infectés par *O. viverrini*. La prévalence d'*O. viverrini* et *S. mekongi* chez les escargots était respectivement de 0,3 % et 0,01 %. La prévalence globale de l'infection par *O. viverrini* chez les poissons d'eau douce était de 26,9 %, les taux d'infection les plus élevés étant observés chez *Hampala dispa* (87,1 %),

Cyclocheilichthys apogon (85,7 %) et *Puntius brevis* (40,0 %). L'analphabétisme et un statut socioéconomique inférieur augmentaient le risque d'infection par *O. viverrini*, alors que les personnes de 10 à 16 ans et possédant des latrines à la maison étaient moins susceptibles d'être infectées. Les chiens et les chats domestiques qui avaient consommé du poisson cru étaient significativement et positivement associés à l'infection par *O. viverrini* des membres du ménage. Concernant *S. mekongi*, les enfants de moins de 9 ans étaient exposés de manière significative à cette infection, par rapport aux groupes plus âgés.

Conclusions: Il y a un besoin urgent de concevoir et de mettre en place une intervention intégrée de lutte contre les helminthes dans les îles du Mékong dans le sud de la RDP Lao. Compte tenu de la transmission très dynamique de *S. mekongi*, *O. viverrini*, des STH et du multiparasitisme étendu, une distribution annuelle massive de médicaments est justifiée, conjointement avec des modifications environnementales, une éducation sanitaire et un meilleur accès à l'eau potable et à un assainissement adéquat pour consolider le contrôle de la morbidité et se diriger vers l'élimination.

Translated from English version into French by CBMtranslations, through

TRANSLATORS
WITHOUT BORDERS

Передача *Opisthorchis viverrini (Беличьей двуустки)*, *Schistosoma mekongi (Шистосомы Меконга)* и геогельминтов на островах дельты реки Меконг, на юге Лаосской Народно-Демократической Республики

Ютанаван Вонгачак (Youthanavanh Vonghachack), Петер Одерматт (Peter Odermatt), Кеока Тайсаявонг (Keoka Taisayyavong), Супхан Фонсават (Souphanh Phounsavath), Конгсап Аккавонг (Kongsap Akkavong), Соппоу Сайасон (Somphou Sayasone)

Краткое изложение содержания

Справочная информация: Уровень распространения *Opisthorchis viverrini (Беличьей двуустки), Schistosoma mekongi (Шистосомы Меконга)* и геогельминта (STH) остается высоким в Лаосской Народно-Демократической Республике (ЛНДР), несмотря на предпринятые властью усилия, которые включают в себя массовую раздачу медикаментов, образовательные и коммуникационные кампании. Необходим новый подход для борьбы с гельминтами.

Методология: исследование в сфере экоздоровья было проведено на двух островах дельты реки Меконг в Южном Лаосе. Демографические и поведенческие данные были собраны с помощью анкет. Были исследованы человеческие и животные испражнения. Улитки *Bythynia (Битиния)* spp. и *Neotricula aperta* были исследованы с использованием излучения. Пресноводная рыба была исследована с помощью техники расщепления. Многомерный анализ случайных уровней факторов был использован для поиска факторов риска, связанных с глистовой инфекцией.

Основные данные: Показатели заражения человека *O. viverrini (Беличьей двуусткой),* анкилостомой, *S. mekongi (Шистосомой Меконга), Trichuris trichiura (Власоглавом)*, *Ascaris lumbricoides (Человеческой аскаридой)* и *Taenia (Бычьей цепенью)* соответствовали 60,7%, 44,1%, 22,2%, 4,1%, 0,6% и 0,1%. Тяжелые инфекции составляли 4,2%, 3,6% и 1,8% с *O. viverrini (Беличьей двуусткой)*, *S. mekongi (Шистосомой Меконга)* и анкилостомой, соответственно. Инфекции *O. viverrini (Беличьей двуустки)* и *S. mekongi (Шистосомы Меконга)*

были у 25,0% и 14,7% собак и котов, соответственно. У 53,1% исследованных котов была инфекция *O. viverrini (Беличьей двуустки)*. Уровень распространения *O. viverrini (Беличьей двуустки)* и *S. mekongi (Шистосомы Меконга)* в улитках составил 0,3% и 0.01%, соответственно. Общая распространенность инфекции *O. viverrini (Беличьей двуустки)* в пресноводной рыбе была 26,9%, с самыми высокими показателями заражения в *Hampala dispar* (87,1%)*,* *Cyclocheilichthys apogon* (85,7%) и *Puntius brevis* (40.0%). Неграмотность и низкий социально-экономический статус повысил риск инфекции *O. viverrini (Беличьей двуусткой)*, в то время, как подростки в возрасте 10 – 16 лет и обладатели уборных в домах были менее склонны к заражению. Домашние собаки и коты, которые потребляли сырую рыбу, были значительно и положительно связаны с инфекцией *O. viverrini (Беличьей двуустки)* членов домохозяйства. Что касается *S. mekongi (Шистосомы Меконга)*, дети до 9 лет были значительно подвержены этой инфекции, по сравнению с более старшими возрастными группами.
Выводы: Существует неотложная необходимость разработки и осуществления комплексного вмешательства для борьбы с гельминтами на островах дельты реки Меконг в южной Лаосской НДР. Учитывая высоко-динамичную передачу *S. mekongi (Шистосомы Меконга), O. viverrini (Беличьей двуустки)*, STH и расширенный мультипаразитизм, ежегодная массовая раздача медикаментов оправдана наряду с изменениями окружающей среды, санитарным просвещением и улучшенным доступом к чистой воде и адекватными санитарными условиям для укрепления контроля за распространением болезней до их полного уничтожения.

Translated from English version into Russian by Viktorija Putneva, through

TRANSLATORS
WITHOUT BORDERS

Transmisión de *Opisthorchis viverrini*, *Schistosoma mekongi* y helmintos transmitidos por el suelo en las islas del Mekong, sur de Laos (PDR)

Youthanavanh Vonghachack, Peter Odermatt, Keoka Taisayyavong, Souphanh Phounsavath, Kongsap Akkavong, Somphou Sayasone

Resumen

Antescedentes: Prevalencia de *Opisthorchis viverrini, Schistosoma mekongi* y helmintos transmitidos por el suelo (STH, por su siglas en inglés) sigue siendo alta en la República Democrática Popular Lao (PDR, por sus siglas en inglés), pese a los esfuerzos de control incluyendo la administración de medicamentos en masa drogas, así como campañas educativas y de comunicación Se requieren nuevos enfoques para avanzar en el control de helmintos.
Metodología: se realizó un estudio de ecosalud en dos islas del Mekong en el sur de Laos. Se recogieron datos demográficos y de comportamiento mediante cuestionario. Se examinaron depósitos de deposiciones humanas y animales. Se usó la técnica del derramamiento para examinar *Bitinia* spp. y *Neotricula aperta*. Se usó la técnica de la digestión para examinar peces de agua dulc. Se hicieron análisis de efectos aleatorios multivariados para encontrar factores de riesgo asociados con las infecciones por helmintos.
Resultados principales: las tasas de infección humana con *O. viverrini,* anquilostoma, *S. mekongi, Trichuris trichiura*, *Ascaris lumbricoides* y *Taenia* spp. fueron del 60.7 %, 44.1 %, 22.2 %, 4.1 %, 0.6 % y 0.1 %, respectivamente. EL 4.2 %, 3.6 % y 1.8 % de las infecciones de intensidad severa correspondieron a *O. viverrini*, *S. mekongi* y anquilostoma, respectivamente. *Las tases de infecciones por O. Viverrini* y *S. mekongi* entre perros y gatos fueron del 25.0 % y 14.7 %,

respectivamente. El 53.1 % de los gatos examinados estaban infectados con *O. viverrini*. La prevalencia de *O. viverrini* y *S. mekongi* en caracoles fue del 0.3 % y 0.01 %, respectivamente. En general, la prevalencia de infección por *O. viverrini* en peces de agua dulce fue del 26.9 %, con las mayores tasas de infección encontradas en las especies *Hampala dispa* (87.1 %), *Cyclocheilichthys apogon* (85.7 %) y *Puntius brevis* (40.0 %). El analfabetismo y el nivel socioeconómico más bajo aumentaron el riesgo de infección por *O. viverrini* , mientras que los individuos de entre 10 y 16 años de edad y que poseían letrinas en su casa eran menos propensos a ser infectados. Los perros y gatos domésticos que consumieron pescado crudo se asociaron de manera significativa y positiva con la infección por *O. viverni* de los miembros del hogar. Con respecto al *S. mekongi*, los niños menores de nueve años estuvieron considerablemente más expuestos a esta infección, en comparación con los grupos de edad mayor.

Conclusiones: es necesario diseñar e implementar una intervención integral para el control de helmintos en las islas del Mekong en el sur de Laos. Teniendo en cuenta la transmisión altamente dinámica de *S. mekongi. O. viverrini*, STH y ultiparasitismo extendido, la administración anual en masa de medicamentos está garantizada junto con modificaciones ambientales, educación para la salud y un mejor el acceso al agua potable y saneamiento adecuado para consolidar el control de la morbilidad y avanzar hacia la eliminación.

Translated from English version into Spanish by Astrid Walter Medina, through

TRANSLATORS
WITHOUT BORDERS
